# Nosocomial infection of CCHF among health care workers in Rajasthan, India

**DOI:** 10.1186/s12879-016-1971-7

**Published:** 2016-11-03

**Authors:** Pragya D. Yadav, Deepak Y. Patil, Anita M. Shete, Prasad Kokate, Pulkit Goyal, Santosh Jadhav, Sanjeev Sinha, Divya Zawar, Surendra K. Sharma, Arti Kapil, D. K. Sharma, Kamlesh J. Upadhyay, Devendra T. Mourya

**Affiliations:** 1 National Institute of Virology, 20-A, Dr. Ambedkar Road, Pune, Maharashtra Pin Code: 411001 India; 2 Goyal Hospital and Research Center, Jodhpur, Rajasthan India; 3 All India Institute of Medical Sciences, New Delhi, India; 4 B J Medical College, Ahmadabad, Gujarat India

**Keywords:** CCHF, Tick, Virus, Gujarat, RT-PCR, Rajasthan

## Abstract

**Background:**

Ever since Crimean-Congo hemorrhagic fever [CCHF] discovered in India, several outbreaks of this disease have been recorded in Gujarat State, India. During the year 2011 to 2015 several districts of Gujarat and Rajasthan state (Sirohi) found to be affected with CCHF including the positivity among ticks and livestock. During these years many infected individuals succumbed to this disease; which subsequently led to nosocomial infections. Herein, we report CCHF cases recorded from Rajasthan state during January 2015. This has affected four individuals apparently associated with one suspected CCHF case admitted in a private hospital in Jodhpur, Rajasthan.

**Case presentation:**

A 30-year-old male was hospitalized in a private hospital in Jodhpur, Rajasthan State, who subsequently had developed thrombocytopenia and showed hemorrhagic manifestations and died in the hospital. Later on, four nursing staff from the same hospital also developed the similar symptoms (Index case and Case A, B, C). Index case succumbed to the disease in the hospital at Jodhpur followed by the death of the case A that was shifted to AIIMS hospital, Delhi due to clinical deterioration. Blood samples of the index case and Case A, B, C were referred to the National institute of Virology, Pune, India for CCHF diagnosis from the different hospitals in Rajasthan, Delhi and Gujarat. However, a sample of deceased suspected CCHF case was not referred. Subsequently, blood samples of 5 nursing staff and 37 contacts (Case D was one of them) from Pokhran area, Jaisalmer district were referred to NIV, Pune.

**Conclusions:**

It clearly indicated that nursing staff acquired a nosocomial infection while attending the suspected CCHF case in an Intensive Care Unit of a private hospital in Jodhpur. However, one case was confirmed from the Pokhran area where the suspected CCHF case was residing. This case might have got the infection from suspected CCHF case or through other routes. CCHF strain associated with these nosocomial infections shares the highest identity with Afghanistan strain and its recent introduction from Afghanistan cannot be ruled out. However, lack of active surveillance, unawareness among health care workers leads to such nosocomial infections.

## Background

Crimean-Congo hemorrhagic fever virus [CCHFV] belongs to the family *Bunyaviridae*, genus *Nairovirus* which cause deadly viral hemorrhagic fever (VHF). The disease has a case fatality rate of up to 80 % in humans [1, 2]. Humans get the infection by tick bites or contact with the blood or body fluids of infected animals or nosocomial infections. The disease has been reported worldwide from Africa, Southeast Europe, Middle East and Asia [3, 4]. Apparently, nosocomial transmission of CCHFV among healthcare workers has been reported from various countries, including India [5–9]. After its confirmation in India, sporadic cases and outbreaks of CCHF were reported during the year 2011–2015 from different districts of Gujarat State (Ahmadabad, Amreli, Patan, Surendranagar, Kutch, Kheda, Aravali, Mehsana and Morabi) and Rajasthan state (Sirohi) of India [10–14]. Serosurvey of CCHF among livestock from 15 districts of Gujarat state and further in 22 states and one union territory of the country showed IgG antibody positivity [15, 16]. This marks the prevalence of CCHF in Gujarat State and other parts of the country. Recently, CCHF case has also been reported from Moradabad, Uttar Pradesh state. In March 2014, the first human case of CCHF has been reported from the Sirohi district of Rajasthan State bordering Gujarat State [17]. Furthermore, seropositivity of CCHF among livestock has been reported from the same area in 2011 [12]. This indicated the risk of CCHF outbreak in Rajasthan State. Herein, we report the confirmation of nosocomial infection of CCHF among health care workers in Rajasthan, India.

## Case presentation

In the second week of January 2015, a suspected case of CCHF; resident of Pokhran area, Jaisalmer district of Rajasthan State was admitted to a private hospital, Jodhpur, Rajasthan State. Eventually, the patient succumbed to death in the hospital. The four nursing staff attending the suspected CCHF case in an Intensive care unit (ICU) developed hemorrhagic manifestations on 17th January 2015. On 18th January, one of the nursing staff (Index case) died in the hospital in Jodhpur. Due to clinical deterioration, one of the nursing staff (Case A) was airlifted to All India Institute of Medical Sciences [AIIMS] hospital, New Delhi, on 20th January; where Case A also succumbed to the disease on 21st January [18]. The other two nursing staff (Case B & Case C) was shifted to Apollo hospital in Ahmadabad, Gujarat State on 19th January 2015. They were under observation in isolation and further survived. The death of the patient (Case A) in AIIMS hospital, New Delhi caused fear of Ebola virus disease in India and this news created panic in public.

The blood and serum samples of four nursing staff were referred to NIV, Pune, India from different hospitals located in Jodhpur (Index case), New Delhi (Case A) and Gujarat (Case B & C) for CCHF diagnosis on different days between 18 and 21 January 2015. However, the sample of deceased suspected CCHF case admitted in a private hospital in Jodhpur was not referred. All these cases were apparently associated with a private hospital in Jodhpur. CCHF virus-specific Real time RT-PCR, RT-PCR, sequencing and anti-CCHF IgM ELISA (Vectocrimean-CHF-IgM ELISA kit) were performed on the referred samples as described earlier [12]. All 12 individuals who were involved in nursing of the index case in a private hospital in Jodhpur were administered with antiviral Ribavirin as supportive treatment. Due to alertness and proper barrier nursing practices, secondary cases of CCHF could be avoided.

The sources reportedly said that suspected CCHF case was from Pokhran area, Jaisalmer district of Rajasthan State, seems to be the source of nosocomial infection [19]. Based on this suspicion, the local public health authorities in that area initiated contact tracing. Contact cases were defined as all people who either had close contact with the confirmed case/primary case in travel, community contact, household settings and patients visit/stay in a hospital. On 27th January, blood samples of 5 nursing staff and 37 contacts (Case D was one of them) living in 50 houses around the residence of suspected CCHF case were withdrawn and referred to NIV, Pune for CCHF diagnosis. A sample of the individual who shared the accommodation with an index case was also referred to NIV, Pune.

### Clinical presentation and laboratory findings

The suspected CCHF case presented in a private hospital, Jodhpur, Rajasthan State with typical hemorrhagic manifestations. All four nursing staff (Index case, Case A, B, C) showed increased levels of the biochemical parameter and abnormal hematological findings. All of them had a platelet count lower than 150.000/mm^3^ and higher levels of Aspartate transaminase (AST), Alanine transaminase (ALT), Creatine kinase (CK) and Lactate dehydrogenase (LDH) than the normal values. Laboratory hematological analysis showed lower WBC, hemoglobin [Hb], hematocrit [Hct], whereas Prothrombin time (PT) values were significantly higher (Table 1). However, the clinical details of Case D were not available.

**Table 1:** Laboratory data on biochemical and hematological parameters in suspected CCHF cases during admission to hospital

Test	Unit/Reference	Index case (NIV151175)	Case A (NIV151149)	Case B (NIV151162)	Case C (NIV151165)
WBCs	4000–10000/CMM	12100	13600	5300	2430
Hb	12–16	15.5	9.1	14.3	14.5
Platelet count	150000–500000/CMM	26000	8000	60000	21000
Lymphocytes	20–45 %	ND	16 %	55 %	60 %
Alanine transaminase [ALT)	9–52 U/L	162	102	166	1146
BUN/Urea	14–36 mg/dl	137/48	120	*NA	*NA
Serum Creatinine	0.7 to 1.3 mg/dL	4.98	4.13	0.6	0.9
Aspartate transaminase [AST)	14–36 U/L	114	95	285	2638
Serum bilirubin [Total)	0.2–1.3 mg/dl	3.80	1.88	0.32	1.87
Serum billirubin [Direct)	0–0.3 mg/dl	2.87	0.95	*NA	*NA
Serum alkaline phosphatase	38–120	553	89	*NA	*NA
PT	12–13 Sec	23	18	*NA	*NA
Total protein	6–8.3 gm/dL	6.31	6.92	*NA	*NA

*NA: The values of biochemical parameters were not available. The detail biochemical and hematological parameter of Case D

All four samples (Index case & Case A, B, C) were found to be positive for CCHF viral RNA, by Real time RT-PCR, RT-PCR, sequencing and anti-CCHF IgM antibodies by ELISA (Table 2). Moreover, samples of 5 nursing staff were found to be negative for CCHF, however, one contact (Case D) of the 37 contacts screened from Pokhran area, Jaisalmer district was found to be positive for CCHF viral RNA and anti-CCHF IgM antibodies. However, it’s difficult to predict, whether case D was a contact of a suspected CCHF case or he might have got the infection via the same route as a suspected CCHF case. A sample of the individual who shared the accommodation with index case was found to be negative for CCHF.

**Table 2:** Details of CCHF diagnosis on referred suspected CCHF cases/contact cases

Sr.No.	NIV No.	Case detail	Age/Gender	Location/Hospital	State	Ct value of CCHFV Real-time RT- PCR	CCHFV IgM ELISA	Recovery status	GenBank Accession numbers of the sequences for S, L, M gene
1	NIV 151175	Index case (Nursing staff: contact of suspected CCHF case)	21 year/Male	Private hospital, Jodhpur	Rajasthan	26	Positive	Died	S (KT384396), L (KT384386), M (KT384394)
2	NIV 151149	Case A (Nursing staff: contact of suspected CCHF case)	30 year/Male	Admitted to AIIMS hospital, Delhi from Jodhpur	Delhi	25	Positive	Died	S (KT384401), L (KT384384), M (KT384403)
3	NIV 151162	Case B (Nursing staff: contact of suspected CCHF case)	29 years/Male	Admitted to Apollo hospital Gandhi Nagar	Gujarat	36	Positive	Survived	NA
4	NIV 151165	Case C (Nursing staff: contact of suspected CCHF case)	25 years/Male	Admitted to Apollo hospital Gandhi Nagar	Gujarat	31	Positive	Survived	L (KT384385)
5	NIV 1510241	Case D (Contact of suspected CCHF case at residence)	-/Male	Pokhran, Jaisalmer	Rajasthan	37	Positive	Survived	NA

### Phylogenetic analysis of CCHF confirmed cases from Jodhpur, Rajasthan State

All five CCHF positive human serum samples (Index case & Case A, B, C, D) were amplified by RT-PCR using specific primers for complete S and partial M and L segment using earlier published primers [20]. Large fragments of M and L could not be sequenced due to less quantity of samples. CCHF Viral RNA could not be amplified from the samples of Case B, C and D because of low viremia (high Ct values >30). However, partial L gene sequencing of Case D could be done. Sequence alignment and phylogenetic tree construction using the neighbor-joining algorithm with 1000-bootstrap replicates was done using MEGA v6.0 software. For comparison and phylogenetic analysis, earlier reported globally representative CCHFV sequences were downloaded from GenBank. P-distances were used for calculating pair wise nucleotide and amino acid sequence identities. To compare the CCHF divergence in Indian CCHF strains, representative CCHF sequences of different outbreaks from Gujarat and Rajasthan states were also included.

Comparison of the current CCHF sequence from Jodhpur revealed that the S gene (GenBank accession no. KT384401) differed from Amreli 2013, Surendranagar 2013, Kutch 2014 and Kutch 2015 sequences by 9.2, 9.1, 10 and 10.4 % at nucleotide level and 2.7, 2.3, 1.7 and 1.9 % at amino acid level respectively. M gene (GenBank accession no. KT384403) showed 7.3, 7.1, 7.7 and 6.9 % difference at nucleotide level and 3.7, 3.2, 4 and 2.8 % at amino acid level. Similarly the L gene (GenBank accession no. KT384384) showed 2.8, 1.6, 1.4, 1.5 % difference at nucleotide level and 2.2, 0.3, 0.5 and 0.5 % at amino acid level (Table 3).

**Table 3:** Divergences between the CCHF virus strains from Jodhpur Russia and related CCHF viral strains

NIV151175_Jodhpur 2015 [GeneBank Accession no. S (KT384396), M (KT384394), L (KT384386)]							
CCHF virus strains	GenBank Accession no.	S gene		M gene		L gene	
		% NT	% AA	% NT	% AA	% NT	% AA
NIV1312492_Amreli_2013	S (KT384399), M (KT384392), L (KT384390)	9.2	2.7	7.3	3.7	2.8	2.2
NIV1312358_Surendranagar_2013	S (KT384398), M (KT384391), L (KT384389)	9.1	2.3	7.1	3.2	1.6	0.3
NIV149210_Kutch_2014	S (KT724950), M (KT384393), L (KT384383)	10.0	1.7	7.7	4.0	1.4	0.5
NIV155249_Kutch_2015	S (KT384402), M (KT384395), L (KT384387)	10.4	1.9	6.9	2.8	1.5	0.5
Tajikistan_1990	S (AY049083), M (AY179962), L (AY720893)	8.8	2.3	11.8	8.7	11.4	2.6
Afghanistan_2009	S (HM452305), M (HM452306), L (HM452307)	0.6	0.4	1.4	0.9	1.8	0.7

Further, an analysis showed maximum nucleotide identity of 99.4 %, 98.2 %, 98.6 for S > L > M segments and at amino acid level 99.6, 99.3 and 99.1 % respectively with Afghanistan strains [GenBank accession no. S (HM452305), L (HM452307), M (HM452306)]. Notably, it had less homology with Indian and Tajikistan strains.

**Fig. 1:**
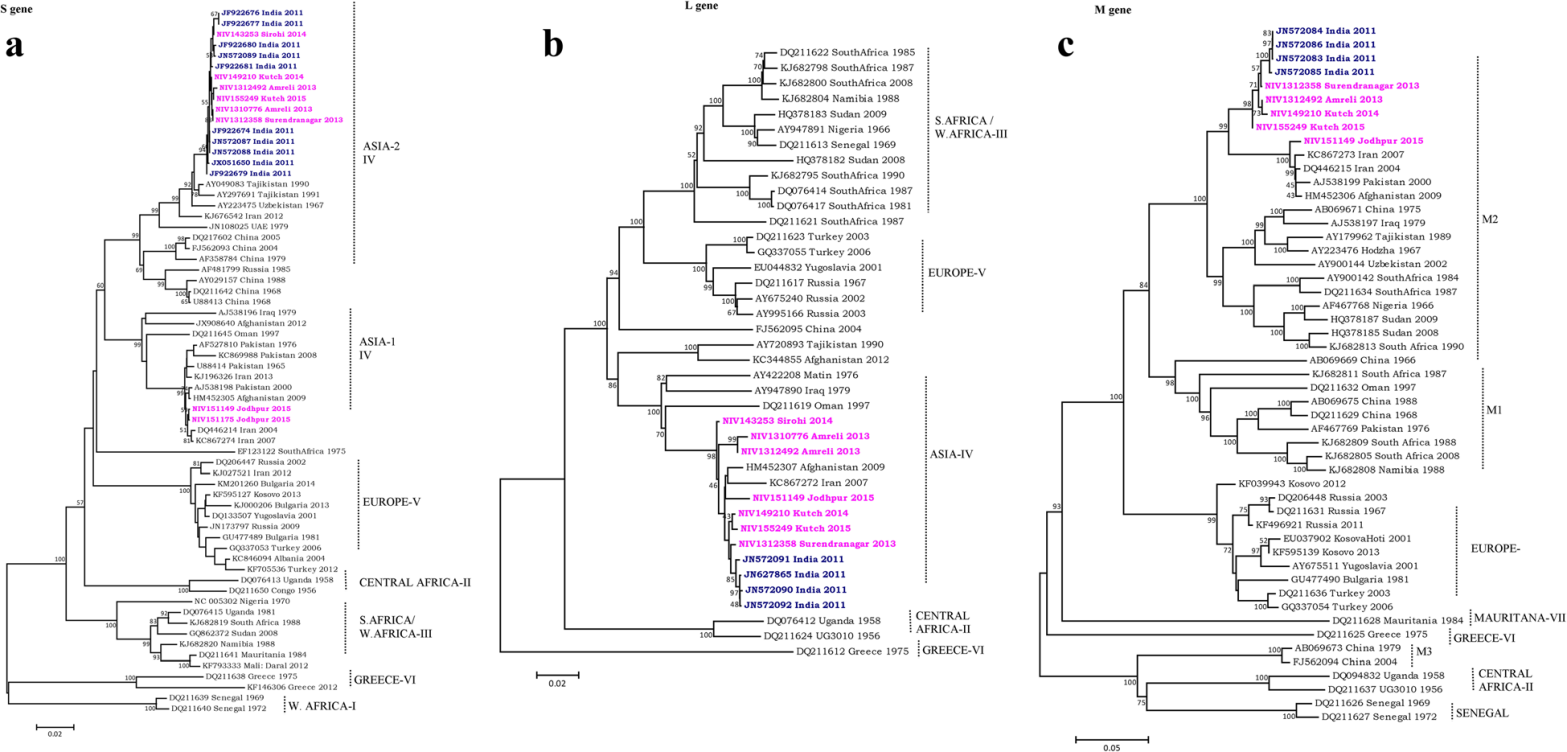
**a**-**c** Phylogenetic tree of CCHF sequences from Rajasthan, India and other known sequences of CCHF virus (S, L and M segment) from Gen Bank

Phylogenetic analysis of the complete S segment demonstrated that the Indian isolates showed maximum relatedness with the Asia-1 sub-group of group IV isolates. Sequences from Jodhpur (2015) showed a close association with isolates from Afghanistan and Iran strain; while earlier Indian CCHF sequences showed a close homology with Tajikistan, Uzbekistan and the Chinese isolates which form the Far East Asia (Asia-2) subgroup [20]. The highest nucleotide sequence identity of CCHF sequence of Jodhpur 2015 was seen with CCHF sequences from Afghanistan and Iran. Similar results were found during phylogenetic analysis of L segment. Phylogenetic comparison of the M segments showed that Indian isolates belonged to the M2 group however the M segment sequences have also shown highest identity with CCHF sequences from Afghanistan and Iran (Fig. 1a-c).

Genetic analysis suggests some genetic exchanges might have occurred in Asia region strains. The data revealed that current CCHF viral strain responsible for this outbreak is different than earlier and its recent introduction from Afghanistan cannot be ruled out.

## Conclusion

Since the first nosocomial outbreak confirmation in the year 2011, Gujarat State of India is known to be endemic for CCHF. Its confirmation in human, ticks and seropositivity among livestock from different districts of Gujarat has been reported in recent years [10–17]. Gujarat has witnessed many outbreaks either in focal or nosocomial form. The risk always existed for Rajasthan as it is the adjoining state of Gujrat and it was evident by confirmation of first CCHF case in Sirohi district and seropositivity of CCHF among livestock [16, 17]. Due to common geographical boundaries, there might have been a transmission of the virus by different means to apparently new area from an endemic region of Gujarat State to Rajasthan. All CCHF viral sequences derived from ticks, human or animal during different outbreaks from Gujarat State during last five years (2011–2015) showed high identity at nucleotide and amino acid level with CCHF sequences of Ahmadabad, Gujarat State (the year 2011) and Sirohi district of Rajasthan State (the year 2014) [12]. These CCHF strains from India were a re-assortment of Tajikistan and Afghanistan strains.

**Fig. 2:**
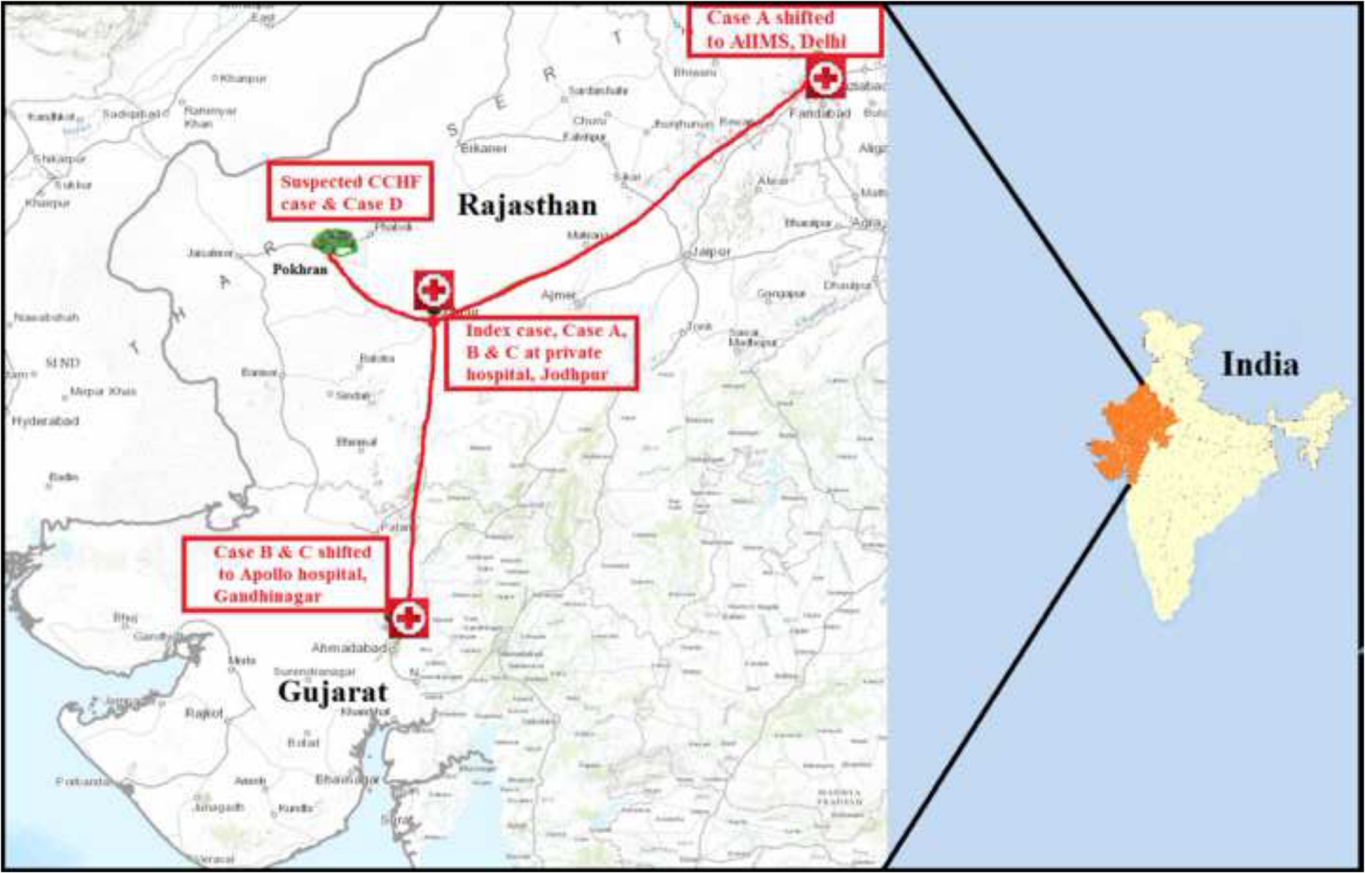
Pictorial presentation of nosocomial outbreak of CCHF in Rajasthan State, India

Herein, we report confirmed cases of CCHF from Rajasthan State of India (Fig. 2). CCHF strain associated with these nosocomial infections was found to be phylogenetically similar to Afghanistan strain (2009) of CCHF virus for all three segments (S, M, and L) and seems to be derived from Afghanistan.

The source of infection in Index case & Case A, B, C seems to be a nosocomial infection, as all of them were involved in nursing suspected CCHF case in a private hospital, Jodhpur. None of them reported any direct contact with domestic animals. However, case D was a resident of the same village as suspected CCHF case. This case might have got the infection through close contact; however, the probability of getting the infection through other routes can’t be ruled out due to the rural setting and socio-economic behavior.

Initially, these suspected viral hemorrhagic fever (VHF) cases created fear of Ebola virus disease imported from African countries and causing nosocomial infection; however, differential diagnosis helped in ruling out this possibility. These emphasize that the clinicians should look for the epidemiological history of hemorrhagic fever cases and consider CCHF in their differential diagnosis in the endemic region. Dengue should be ruled out during the preliminary screening of VHF cases. In the past, other CCHF case reports have confirmed that levels of AST, ALT, PT, PTT and LDH were significantly higher among severe CCHF cases reported from India and globally during different outbreaks. Besides the laboratory findings, melena and hematemesis were also defined as the parameters for the severity [21]. This suggests the value of the blood count and the biochemical tests in the early diagnosis of the CCHF.

In conclusion, this clearly seems to be a nosocomial outbreak of CCHF in a private hospital in Jodhpur, Rajasthan State. Health-care workers are an important risk group as evidenced by the large number of nosocomial infections often associated with CCHF outbreaks [6–10, 13]. Infected patients should be isolated and subjected to barrier nursing techniques. Health-care workers should mandatorily wear minimum essential personal protective equipment while attending the patients or while performing any allied procedures. It is interesting to note that no tertiary cases were reported in AIIMS hospital, Delhi where positive CCHF case was handled. This could be due to effective biosafety measures followed during nursing the case and administration of Ribavirin to all potential hospital contacts.

State government of Gujarat has made efforts for the cautiously handling of suspected CCHF cases. High level of preparedness plan was implemented in India at the country level during Ebola outbreak situation in the year 2014. However, CCHF belongs to high-risk group of disease and should also be taken at priority. Moreover, we emphasize the need for syndrome-based surveillance of VHF cases for CCHF and strict infection control measures in the hospital environment in Rajasthan State urgently.

## Abbreviations

AIIMS: All India Institute of Medical Sciences
ALT: Alanine transaminase
AST: Aspartate transaminase
CCHF: Crimean-Congo hemorrhagic fever
CK: Creatine kinase
Hb: Hemoglobin
Hct: Hematocrit
ICU: Intensive care unit
LDH: Lactate dehydrogenase
NIV: National Institute of Virology
PT: Prothrombin time
Real-time RT-PCR: Real time reverse transcriptase polymerase chain reaction
VHF: Viral hemorrhagic fever
